# Predictors of Caregiver Burden in Delirium: Patient and Caregiver Factors

**DOI:** 10.1093/geroni/igaa057.818

**Published:** 2020-12-16

**Authors:** Patricia Tabloski, Franchesca Arias, Nina Flanagan, Tamara Fong, Eva Schmitt, Richard Jones, Thomas Travison, Sharon Inouye

**Affiliations:** 1 Boston College, Sudbury, Massachusetts, United States; 2 Marcus Institue for Aging Rsearch, Hebrew Senior Life, Boston, Massachusetts, United States; 3 Binghamton University, Vestal, New York, United States; 4 Institute for Aging Research / Hebrew SeniorLife, Boston, Massachusetts, United States; 5 Harvard Medical School, Boston, Massachusetts, United States; 6 Brown University, Providence, Rhode Island, United States; 7 Harvard University, Boston, Massachusetts, United States

## Abstract

Delirium — an acute disorder of attention and cognition — is a common, life-threatening and costly syndrome occurring frequently in older hospitalized persons. The unexpected, rapid, and volatile nature of delirium can be difficult for family caregivers to experience and may contribute to subjective feelings of distress (i.e. “delirium burden”). The aim of this study was to examine whether pre-admission patient characteristics or patient-caregiver relationship and living arrangements were associated with caregiver burden as measured by the delirium burden scale for caregivers (DEL-B-C; score 0-40, higher score is more burden). Our sample consisted of 208 older adults and their caregivers from the Better Assessment of Illness (BASIL) study, an ongoing prospective, observational study of surgical and medical patients ≥70 years old; 22% of patients experienced delirium by the Confusion Assessment Method (CAM) and the average DEL-B-C score was 7.9, 95% CI(6.95-8.88). Results indicated that neither patient-caregiver relationship and living arrangement or patient factors including pre-admission pain, sleep disturbance, or new onset incontinence were significantly correlated with delirium-related caregiver burden. However, DEL-B-C scores were significantly higher in caregivers of patients with any ADL impairment (mean 8.5 vs. 5.2, p = .016) during hospitalization although none of the individual functional deficits alone were statistically significant. This finding suggests that the association of ADL impairment and DEL-B-C scores is not driven by a single functional domain. Future studies are needed to further understand how caregiver characteristics and patient factors occurring before and during hospitalization contribute to caregiver burden after the occurrence of delirium.

